# Retrospective multicentre evaluation of common calcaneal tendon injuries in 66 cats. Part 2: treatment, complications and outcomes

**DOI:** 10.1177/1098612X221131224

**Published:** 2023-01-27

**Authors:** Thomas C Häußler, Matthias Kornmayer, Miriam Scheich, Andreas Fischer, Christian J Feichtenschlager, Thomas Rohwedder

**Affiliations:** 1 Department of Veterinary Clinical Sciences, Small Animal Clinic – Surgery, Justus-Liebig-University, Giessen, Germany; 2 Clinic for Small Animal Surgery and Reproduction, Ludwig-Maximilians-University, Munich, Germany; 3 Small Animal Clinic Hofheim, Hofheim, Germany; 4 Small Animal Clinic Kalbach, Kalbach, Germany; 5 Small Animal Clinic, Free University Berlin, Berlin, Germany

**Keywords:** Common calcaneal tendon, Achilles tendon, tendinopathy, rupture

## Abstract

**Objectives:**

The aims of the second part of this retrospective multicentre study were to describe the surgical techniques used in the treatment of common calcaneal tendon (CCT) injuries, and evaluate the short- and long-term outcomes and complications.

**Methods:**

The medical records of five different small animal referral centres and veterinary teaching hospitals between 2010 and 2020 were reviewed. Surgical vs conservative treatment was evaluated. Treatment type, type of postoperative immobilisation, and short- and long-term outcomes and complications were recorded. Minor complications were defined as not requiring surgical intervention. Long-term outcome was evaluated by an owner questionnaire.

**Results:**

Sixty-six cats met the inclusion criteria. Mean time to surgery was 9.6 days (range 0–185). Most cats (83.3%) were treated surgically. Regardless of treatment modality, all limbs were immobilised for a mean time of 48.2 days (range 2–98). For 63 cats that had the temporary tarsal joint immobilisation technique recorded, a transarticular external skeletal fixator (ESF; 57.1%) or a calcaneotibial screw (33.3%) were used most commonly. The method of immobilisation had a notable, although non-significant, influence on the occurrence of short-term complications, with most complications being reported for the transarticular ESF group. The total short-term complication rate was 41.3%, the minor complication rate was 33.3% and the major complication rate was 7.9%, with pin tract infections being the most commonly occurring minor complication. Three cats (6%) had a total of four major complications over the long term. Most cats (86%) were free of lameness at the long-term evaluation, with an overall successful clinical long-term outcome of 84.9%, according to the owner questionnaire. Cats with traumatic injuries and injuries treated surgically had higher questionnaire scores than those with atraumatic injuries and those treated conservatively.

**Conclusions and relevance:**

Outcome was generally good in cats with CCT injury, irrespective of the type of treatment. Complications included a high proportion of minor complications associated with the technique of tarsal joint immobilisation. ESF frames were more commonly involved in complications than other techniques. Surgically treated cats had a slightly better long-term outcomes.

## Introduction

Common calcaneal tendon (CCT) injuries in cats are considered uncommon in the veterinary literature.^1–3^ The few existing reports provide limited information about treatments and outcomes in cats.^1,3^ Usually, surgical repair of the tendon with additional temporary tibiotarsal joint immobilisation is recommended. Several surgical techniques have been described.^4–11^

Meutstege introduced a classification system of canine CCT injuries^6^ that has also been used in cats.^3^ A complete tear of all CCT parts is described as type I injury. Musculotendinous ruptures are type IIa lesions. Ruptures of the Achilles tendon (AT) with intact paratenon are type IIb lesions. Type IIc lesions refer to gastrocnemius tendon (GT) avulsions without involvement of the tendon of the superficial digital flexor muscle (SDFT).^6^ Type III lesions include tendinosis or peritendinitis. An alternative time-dependent classification system was proposed by Reinke et al,^12^ where acute injuries are defined as <2 days between tendon injury and surgical treatment; 2–21 days between injury and surgery define subacute injuries, and >21 days between injury and surgery characterise chronic lesions.^12^ There is only one report in the veterinary literature on the surgical repair of AT injuries in cats that considered the type of injury, surgical technique and immobilisation, as well as the short- and long-term outcomes; however, the study was statistically limited owing to the small number of cases (21 cats).^3^ The objective of the present study was to collect a considerably larger sample size encompassing different surgical and immobilisation techniques to better judge the short- and long-term outcomes in cats with CCT injuries. We hypothesised that surgically treated cats would show better short- and long-term outcomes.

## Materials and methods

### Data acquisition

The data included patients presented to five different small animal clinics in Germany (Free University of Berlin; Ludwig Maximilians University Munich; Justus Liebig University Giessen; Small Animal Clinic Hofheim; Small Animal Clinic Kalbach) between 2010 and 2020. Signalment, body weight (BW), type of injury, type of trauma and time to surgery were recorded. In the first part of the study, we identified and classified CCT injuries according to Meutstege^6^ and Reinke et al^12^ in a study population of 66 cats (see Table 1 in the supplementary material) and evaluated if signalment had any impact on the occurrence and type of CCT injury.^13^ If the fully or partially ruptured CCT had not been sutured but the tarsal joint had been surgically immobilised for a certain time, this was documented as ‘surgical immobilisation without tendon repair’ and was categorised as conservative treatment. Suturing the tendon combined with immobilisation was categorised as surgically treated. The tendon repair technique, different methods of temporary tarsal joint immobilisation, duration of immobilisation, suture pattern, suture material and suture strength were recorded. The use of external coaptation in addition to other immobilisation techniques was documented. Minor and major complications were recorded during short-term evaluation (within 3 months postoperatively) and long-term evaluation (>6 months postoperatively). Minor complications were defined as not requiring surgical intervention. Major complications needed surgical treatment to be managed. The short-term outcome was evaluated by using clinical records and correspondence with the referring veterinarians. The long-term outcome was evaluated by calling the referring veterinarians and via an owner questionnaire.

**Table 1:** Body weight (BW), age and sex distribution of cats in the atraumatic and traumatic groups, and total study population

	Type of injury			
	Atraumatic (n = 21 [31.8%])	Traumatic (n = 25 [37.9%])	Not classified (n = 20 [30.3%])	Total (n = 66)
BW (kg)	5.2 ± 1.5 (3.0–9.0)	4.3 ± 1.1 (1.5–6.4)	4.4 ± 0.7 (3–5.3)	4.6 ± 1.2 (1.5–9.0)
Age (years)	10.4 ± 4.0 (0.5–15.3)	5.2 ± 4.3 (0.6–15.8)	7.5 ± 4.2 (0.5–16.3)	7.5 ± 4.7 (0.5–16.3)
Sex				
FI	1 (4.8)	2 (8)	2 (10)	5 (7.6)
FS	10 (47.6)	13 (52)	11 (55)	34 (51.5)
MC	10 (47.6)	10 (40)	7 (35)	27 (40.9)

Data are presented as mean ± SD (range) or n (%)

FI = female intact; FS = female spayed; MC = male castrated

### Questionnaire

The questionnaire (see the Appendix in the supplementary material), previously published by Cervi et al,^3^ was adjusted for use in a German-speaking country. It consists of 15 Likert-style questions and is comparable to a visual analogue scale that had been evaluated previously.^14^ The owners were instructed on how to complete the questionnaire. Answers were given numeric values from 1 to 5. In most cases, 1 was the lowest (worst) score and 5 the highest (best). However, some items were reverse scored, meaning that the numerical scoring scale runs in the opposite direction. For example, the best result would attract a score of 1 instead of 5, and the worst result would attract a 5 instead of a 1. All reverse-scored questions were corrected prior to evaluation meaning 1 was always the lowest (worst) score and 5 was always the highest (best).

Two questions regarding the play behaviour of the cat prior to injury and postoperatively were summarised to represent the behavioural change during the rehabilitation period (questions 4 and 5 in the owner questionnaire; see the Appendix in the supplementary material). No change in postoperative play frequency was given a score of 3. A decrease in play frequency of 1 or 2 points was given a score of 2 and a decrease of 3 or 4 points was given a score of 1 (worst). An increase in play frequency of 1 or 2 points was given a score of 4 and an increase of 3 or 4 points was scored 5 (best).

The maximum final cumulative score of the questionnaire was 70, which was considered to be the best clinical result. The cumulative score and the percentage of the maximum score were recorded. Some cats had already died when their owners answered the questionnaire. In those cases, the long-term follow-up period was determined as the time between surgical treatment and the date of the cat’s death, provided death was unrelated to the CCT injury or treatment.

### Statistical analysis

The mean ± SD values were calculated for age, BW time between injury and surgery, duration of immobilisation, duration of additional external coaptation during immobilisation or after removal of the immobilisation modality, duration of short- and long-term follow-up, and cumulative scores and percentages of the maximum score for the questionnaire. Short- and long-term complications were noted. Six intact cats were excluded from the evaluation of the influence of sex on complications and outcome due to their low proportion of the total population.

The association between multiple factors and the occurrence of short-term complications, as well as the short-term outcome, were evaluated. These factors included age, BW, sex, type of injury (open vs closed), type of trauma (traumatic vs atraumatic), time to surgery, occurrence of short-term complications, type of immobilisation (external skeletal fixator [ESF] frame vs calcaneotibial screw) and use of external coaptation at any time of treatment. The ‘method of immobilisation’ groups with ESF type II and modified ESF type II were combined and compared with the calcaneotibial screw group. There were not enough cats for statistical analysis in the other immobilisation groups (five cats with external coaptation as primary method of immobilisation and one cat with tendon plating). The final logistic regression model to evaluate the occurrence of short-term complications included only the factors ‘age’, ‘external coaptation yes/no’ and ‘type of immobilisation’, with 53 cats (excluding intact cats and cases with incomplete data regarding the ‘time to surgery’). The final model to evaluate the short-term outcome included the independent variables ‘age’, ‘type of injury’ and ‘occurrence of short-term complications’. Statistical models were built as described by Häußler et al.^13^ A *P* value <0.05 was considered to be statistically significant, and *P* <0.001 was considered to be highly statistically significant.

## Results

### Study population

Seventy cats with common calcaneal tendinopathies were identified from the medical records. Four Maine Coon cats were excluded due to increased body size resulting in increased BW without being obese. In total, 66 cats were therefore evaluated (see Table 1 in the supplementary material). Age, BW and sex distribution are shown in Table 1. Injury classification is presented in Table 2.

**Table 2:** Distribution of type of tendon injury according to the Meutstege^6^ classification system in the total study population and traumatic vs atraumatic groups, and distribution of the type of tendon injury according to the time-dependent classification system of Reinke et al^12^

Meutstege classification	Type of injury		
	Atraumatic (n = 21 [31.8%])	Traumatic (n = 25 [37.9%])	Total (n = 66)
I	2 (9.5)	18 (72)	25 (37.9)
IIa	1 (4.8)	–	3 (4.5)
IIb	1 (4.8)	–	1 (1.5)
IIc	15 (71.4)	7 (28)	35 (53)
III	1 (4.8)	–	1 (1.5)
Not defined	1 (4.8)	–	1 (1.5)
Meutstege classification	Time-dependent injury classification		
	Acute (n = 27 [40.9%])	Subacute (n = 34 [51.5%])	Chronic (n = 5 [7.6%])
I	15 (55.6)	11 (32.4)	–
IIa	1 (3.7)	1 (2.9)	1 (20)
IIb	–	–	1 (20)
IIc	11 (40.7)	20 (58.8)	3 (60)
III	–	1 (2.9)	–

Data are presented as n (%)

Twenty cases could not clearly be classified as atraumatic or traumatic in origin

### Treatment

The mean ± SD time from recognition of lameness by the owner to surgery was 9.6 ± 24.5 days (range 0–185). The CCT was sutured in 55/66 cats (83.3%). Eleven cats (16.7%) were classified as receiving conservative treatment (Table 3). In one of the conservatively treated cats (1.5%), the luxated SDFT was sutured in place and the injury to the remaining CCT was treated conservatively with immobilisation only. Regardless of the treatment modality, all limbs were immobilised at the level of the tarsal joint for a mean ± SD time of 48.2 ± 15.5 days (range 2–98). The most commonly used suture material was polydioxanone (n = 49/55 [89.1%]). The most commonly applied suture strength was 2/0 USP (3 metric; n = 39/55 [70.9%]). The suture pattern for tendon repair was available in 54/66 cats (81.8%). In most cases, a locking loop was used for mid-tendon ruptures (n = 26/54 [48.1%]). Cases with avulsion of the GT from the calcaneus (n = 35 [53%]) were treated by drilling one or two mediolateral bone tunnels through the calcaneus and securing the tendon to the bone after apposition, applying modified versions of the locking loop (16/35 cats [45.7%]), Bunnell–Mayer (7/35 cats [20%]) or three-loop pulley suture (2/35 cats [5.7%]).

**Table 3:** Treatment details for 66 cats with common calcaneal tendon injuries

Treatment	
Surgical	55 (83.3)
Conservative	11 (16.7)
Suture material*	
Polydioxanone	49 (89.1)
Polypropylene	3 (5.5)
Polyglactin 910	2 (3.6)
Polyamide	1 (1.8)
Suture strength*	
3/0 USP (2 metric)	10 (18.2)
2/0 USP (3 metric)	39 (70.9)
0 USP (3.5 metric)	5 (9.1)
1 USP (4 metric)	1 (1.8)
Suture pattern†	
Locking loop	26 (48.1)
Bunnell–Meyer	17 (31.5)
Three-loop pulley	3 (5.6)
Combination of patterns	8 (14.8)
Postoperative immobilisation‡	
Transarticular ESF type II	25 (39.7)
Modified transarticular ESF type II	11 (17.5)
Calcaneotibial screw	21 (33.3)
External coaptation (fibreglass cast)	5 (7.9)
Tendon plating + external coaptation	1 (1.6)

Data are presented as n (%)

* Common calcaneal tendon injuries were sutured in 55 cats

† Suture pattern was available for 54 cats

‡ Information on postoperative immobilisation technique was available for 63 cats

ESF = external skeletal fixator

In three cases (4.5%), the immobilisation technique was not recorded (Table 3). In most cats, postoperative immobilisation was achieved by a transarticular ESF (n = 36/63 [57.1%]) or a calcaneotibial screw (n = 21/63 [33.3%]). All ESF frames were constructed with polymethylmethacrylate (PMMA), and tarsal joints were immobilised in 145° extension.

External coaptation in addition to the primary immobilisation technique was noted in five cases with calcaneotibial screws (7.6%) and three cases with a modified transarticular ESF type II (4.5%), with a mean ± SD time of 11.8 ± 6.6 days (range 5–28). Further external coaptation following removal of the primary stabilisation technique was documented in 11 cases (16.7%): after removal of the transarticular ESF in nine cats (13.6%), after removal of a broken calcaneotibial screw in one cat (1.5%) and with tendon plating in one cat (1.5%). The mean ± SD time of further external coaptation was 16.6 ± 9.0 days (range 2–35).

### Complications

Short-term evaluation was available for 63/66 cats (95.5%). The remainder were lost to follow-up. The mean ± SD short-term period was 54.1 ± 16.9 days (range 28–91). Thirty-two minor and major complications occurred in 26/63 cats in the 3-month period (total short-term complication rate of 41.3%; Table 4). Two cats of 66 (3.0%) suffered from two consecutive major complications. No complications occurred in 37/63 cats (58.7%). The mean ± SD time to complication occurrence was 33.3 ± 17.7 days (range 4–90) postoperatively. Minor complications occurred at a mean ± SD time of 34.8 ± 19 days (range 4–90) and major complications occurred at a mean ± SD time of 23.4 ± 14 days (range 12–49).

**Table 4:** Short-term complications in 63 cats

Minor complications*			Major complications^†^	
Pin tract infection		10 (40)	Breakage of the PMMA bar	1 (14.3)
Pin or screw loosening		5 (20)	Re-rupture of the calcaneal tendon	2 (28.6)
Bandage-related issues		5 (20)	Calcaneal apophysiolysis	1 (14.3)
Swelling around the tarsal joint		3 (12)	Severe infection – hindlimb amputation	1 (14.3)
Minor breakage of the PMMA bar‡		2 (8)	Calcaneal fractureESF failure	1 (14.3)1 (14.3)
Transarticular ESF frames (n = 36 [57.1%])				
Minor complications		17 (47.2)		
Pin tract infections		10 (27.8)		
Minor pin loosening		3 (8.3)		
Minor breakage of the PMMA bar		2 (5.6)		
Swelling around the tarsal joint		2 (5.6)		
No minor complications		19 (52.8)		
Calcaneotibial screws (n = 21 [33.3%])				
Minor complications		5 (23.8)		
Minor screw loosening		2 (9.5)		
Bandage-related skin sores		2 (9.5)		
Swelling around the tarsal joint		1 (4.8)		
No minor complications		16 (76.2)		
Complications	Acute injuries (n = 26/63 cats [41.3%])	Subacute injuries (n = 32/63 cats [50.8%])	Chronic injuries (n = 5/63 cats [7.9%])	
Total	14 (53.8)	14 (43.7)	4 (80)	
Minor	12 (46.1) (11 cats)	9 (28.1) (8 cats)	4 (80) (2 cats)	
Major	2 (7.7) (1 cat)	5 (15.6) (4 cats)	–	
None	14 (53.8)	20 (62.5)	3 (60)	

Data are presented as n (%)

* Twenty-five complications in 21/63 cats (33.3%)

† Seven complications in 5/63 cats (7.9%)

‡ Polymethylmethacrylate (PMMA) bar breakage without pin loosening, fixed without anaesthesia

ESF = external skeletal fixator

The method of temporary transarticular immobilisation had a notable but non-significant influence on the occurrence of short-term complications (*P* = 0.078). Seventeen of 25 minor complications (68%) were attributed to the ESF groups, 5/25 (20%) to the calcaneotibial screw and 1/25 (4%) to a single cat with a cast as sole temporary transarticular immobilisation technique (Table 4).

Applying external coaptation at any time point during treatment had a significant influence on the occurrence of short-term complications (*P* = 0.026). This was not influenced by the duration of bandage therapy. Ten of 21 cats (47.6%) with minor complications had external coaptation postoperatively as sole or additional stabilisation or after implant removal. Of those cats, five (50%) showed bandage-related issues during the short-term evaluation (pressure sores at the calcaneus).

The long-term evaluation was documented by telephone calls with the referring veterinarians and via an owner questionnaire. The mean ± SD time of long-term evaluation was 58.5 ± 35.8 months (range 6.1–154). The long-term results were available for 50/66 cats (75.8%). Of these cats, 47 (94%) had no complications and three (6%) had a total of four major complications with a mean ± SD time of occurrence of 7.4 ± 1.7 months (range 6.0–9.2). One cat accounted for a total of four major complications during short- and long-term evaluations (36.4% of all major complications). Owing to the small number of cats with long-term complications, statistical significance could not be determined. The three cats with major long-term complications were spayed females with a mean ± SD age of 3.3 ± 1.5 years (range 1.8–5.3) and a mean ± SD BW of 5.0 ± 0.9 kg (range 3.4–5.6). The mean ± SD time to surgery was 92 ± 70 days (range 16–185). All were treated surgically. Two cats had a Meutstege type IIc injury and one cat had a type IIa injury. The type IIa injury was sutured with 2/0 USP polydioxanone, whereas the type IIc injuries were sutured with 0 USP polydioxanone and polypropylene, respectively. The mean ± SD total time of immobilisation was 47.7 ± 9.1 days (range 38–56). All three cats had a trans-articular ESF and additional external coaptation for 1–2 weeks. Two of them were already noted with at least one minor complication during the short-term evaluation.

The time-dependent classification according to Reinke et al^12^ and corresponding short-term complications are shown in Table 4. In the logistic regression models, the factor ‘time to surgery’ had no influence on the occurrence of short-term complications. BW and obesity did not have a significant influence on the occurrence of short-term complications. Long-term evaluation was available for 20/21 acute cases (95.2%). None presented with complications at the long-term evaluation. Of the subacute cases, 25/34 (73.5%) were evaluated in the long-term period. One cat (4%) had a major complication 9 months after surgery. Two of five chronic cases (40%) had major complications. Comparing conservatively with surgically managed cases, the conservative cases (nine available) showed no complications during the short- and long-term evaluations.

### Outcomes

The short-term outcome within a 3-month period was available for 59/66 (89.4%) cats. For 7/66 (10.6%) cats, an orthopaedic examination with lameness grading at the end of the short-term period was not documented in the medical records. The outcomes are shown in Table 5. In the logistic regression model, age had a significant influence on the short-term outcome (*P* = 0.023). The occurrence of complications during the short-term evaluation interval had a notable but non-significant influence on the short-term outcome (*P* = 0.071). The factors ‘time to surgery’, ‘BW’ and ‘obesity’ had no significant influence on the short-term outcome.

**Table 5:** Short- and long-term outcomes in conservatively* vs surgically managed cases, acute vs subacute vs chronic injuries, atraumatic vs traumatic injuries and whether the cat was obese

Short-term outcome	Lameness grade 0/	Lameness grade 1/	Lameness grade 2/	Lameness grade 3/	Lameness grade 4/
Total population (n = 59)	40 (67.8)	8 (13.6)	10 (16.9)	–	1 (1.7)
Conservatively managed* (n = 9)	7 (77.8)	–	2 (22.2)	–	–
Surgically managed (n = 50)	33 (66)	9 (18)	7 (14)	–	1 (2)
Acute injuries (n = 23)	15 (65.2)	4 (17.4)	4 (17.4)	–	–
Subacute injuries (n = 31)	24 (77.4)	3 (9.7)	3 (9.7)	–	1 (3.2)
Chronic injuries (n = 5)	2 (40)	1 (20)	2 (40)	–	–
Long-term outcome					
Total population (n = 50)	43 (86)	2 (4)	5 (10)	–	–
Conservatively managed* (n = 7)	6 (85.7)	–	1 (14.3)	–	–
Surgically managed (n = 43)	38 (88.4)	1 (2.3)	4 (9.3)	–	–
Acute injuries (n = 20)	17 (85)	–	3 (15)	–	–
Subacute injuries (n = 25)	25 (100)	–	–	–	–
Chronic injuries (n = 5)	2 (40)	1 (20)	2 (40)	–	–
	Mean questionnaire points (maximum 70)		Successful outcome (%)		
Total population (n = 50)	59.4 ± 7.4 (30–70)		84.9		
Atraumatic cases (n = 15)	54.9 ± 9.8 (30–66)		78.5		
Traumatic cases (n = 20)	61.1 ± 6.1 (45–70)		87.3		
Conservatively managed* (n = 7)	57.3 ± 4.9 (51–63)		81.8		
Surgically managed (n = 46)	59.8 ± 7.8 (30–70)		85.4		
Obese (n = 28)	60.1 ± 6.4 (46–68)		85.8		
Acute injuries (n = 29)	59.6 ± 6.7 (45–70)		85.9		
Subacute injuries (n = 36)	61.4 ± 4.5 (49–66)		87.7		
Chronic injuries (n = 4)	49.1 ± 13.2 (30–63)		70		

Data are presented as n (%) or mean ± SD (range)

* Conservatively managed cases included cats without primary tendon repair but with surgical tarsal joint immobilisation

The mean time taken to answer the questionnaire corresponded to the mean time of the long-term evaluation and outcome. Fifty pet owners returned completed questionnaires. Therefore, long-term outcome information was available for 50/66 (75.8%) cats. Cats with traumatic injuries had higher scores than those with atraumatic injuries (Table 5). In cats with long-term complications (n = 3), one cat had persistent grade 1 lameness, one still showed grade 2 lameness and one cat was free of lameness. These three cats reached a maximum score of 47.7/70 on the questionnaire, leading to a successful outcome in 68.1%. Owing to the low number of long-term complications, further statistical evaluation was not possible.

Differentiation of the long-term outcome regarding the time to treatment was performed (Table 6). Twenty of 27 cats with acute injuries (74.1%), 25/34 cats with subacute injuries (73.5%) and all five of the chronic cases were available for long-term evaluation. Short-term and long-term outcomes of conservatively treated cats and those that were surgically immobilised without primary tendon repair are presented in Table 7.

**Table 6:** Long-term outcome differentiated by the duration of injury

Time to surgery	Long-term outcome							
	Grade 0/4 lameness	Grade 1/4 lameness	Grade 2/4 lameness	Grade 3/4 lameness	Grade 4/4 lameness	Mean score (points)	Total successful outcome (%)	Mean time to follow-up (years)
Acute (n = 20)	17 (85)	–	3 (15)	–	–	59.6 ± 6.7 (45–70)	85.9	4.2 ± 2.4 (0.5–9.7)
Subacute (n = 25)	25 (100)	–	–	–	–	61.4 ± 4.5 (49–66)	87.7	5.7 ± 3.4 (0.9–12.8)
Chronic (n = 5)	2 (40)	1 (20)	2 (40)	–	–	49 ± 13.2(30–63)	70	3.8 ± 1.5 (2.0–6.5)

Data are presented as n (%) or mean ± SD (range)

**Table 7:** Injury specifications of the cats that were conservatively treated and those that were surgically immobilised without primary tendon repair

Cat	Breed	Injury type	Trauma type	Meutstege classification type	Short-term outcome lameness grade 0–4	Long-term outcome lameness grade 0–4
1	DSH	Closed	Unknown	IIc	0	0
2	DSH	Closed	Atraumatic	IIc	0	NA
3	DSH	Closed	Unknown	IIc	0	0
4	DSH	Closed	Unknown	IIc	0	0
5	DSH	Closed	Unknown	I	2	2
6	BSH	Closed	Unknown	III	0	NA
7	DSH	Closed	Pinching injury	IIc	0	NA
8	DSH	Closed	Atraumatic	IIc	0	0
9	DSH	Closed	Atraumatic	NA	NA	NA
10	BSH	Closed	Atraumatic	IIc	2	0
11	DSH	Closed	Atraumatic	IIc	NA	NA

DSH = domestic shorthair; BSH = British Shorthair; NA = not available

## Discussion

The results of the present multicentre retrospective study, which evaluated 66 cats with CCT, found a generally good outcome slightly favouring traumatic injuries and primary surgical treatment of the CCT over atraumatic injuries and conservative treatment. The long-term outcome was evaluated by telephoning the referring veterinarian and via an owner questionnaire. Every question was given a score of 1–5 points and the best possible cumulative score was 70 points, meaning a 100% successful outcome. Cats with atraumatic injuries achieved a mean cumulative score of 54.9 vs 61.1 in traumatic cases. With these results in mind, it should be noted that 26% of the atraumatic cases were managed without primary tendon repair and by tarsal joint immobilisation only. In the traumatic group, 96% were managed surgically, a difference that could have contributed to the better long-term outcome. These results may suggest a better outcome for sutured tendons.

For time-dependent classification of AT injuries, we referred to Reinke et al.^12^ Based on the questionnaire, the best long-term outcome was achieved by cats with subacute injuries, followed by those with acute injuries with a mean score of 61.4/70 and 59.6/70, respectively. Cats with chronic injuries achieved a mean cumulative score of 49. Cervi et al determined the success rate by the percentage calculation of the cumulative score of the maximum score.^3^ In doing so, acute, subacute and chronic injuries had repair success rates of 85.9%, 87.7% and 70%, respectively. Of note, 48.1% of acute injuries and 17.6% of subacute injuries were open injuries, whereas 20% of chronic injuries were categorised as open.13 Acute and open injuries might have better outcomes. This could be explained by the earlier presentation of acute cases and the fact that open injuries are more frequently addressed surgically. This assumption has been confirmed by Cervi et al,^3^ who reported better outcomes in cats with full disruption of the AT addressed surgically rather than with conservative treatment. In the logistic regression model evaluating the short-term outcome, the factor injury type (open vs closed) was relevant. Eliminating this factor decreased the value of the calculation. A statistical tendency for closed injuries (and therefore more commonly atraumatic injuries) for a worse short-term outcome and higher residual lameness grades could not be determined in the present study.

The logistic regression model evaluating factors influencing the short-term outcome revealed that age was statistically significant (*P* = 0.023), with juvenile cats tending to have a higher occurrence of residual lameness at the short-term evaluation compared with their adult counterparts. This could be explained by the fact that young cats tended to present with acute traumatic injuries that might be more painful in the short-term, leading to a higher degree of lame patients.

In one case series with three cats (all with type IIc lesions), all were managed conservatively.^1^ With conservative treatment, gastrocnemius muscle contraction will inevitably lead to gap formation between the avulsed tendon ends or the tendon end and the tuber calcanei. Additionally, there is decreased blood supply in the inert tissue of tendons.^15,16^ Therefore, it appears less likely that proper healing of the tendon will occur without surgical treatment. Nevertheless, the conservatively managed cats in our study had a successful long-term outcome (81.8%), based on the questionnaire vs 85.4% for the surgically addressed cases (Table 5). Considering these data, it could be concluded that primary surgical treatment of the AT might not be necessarily warranted, as proposed by Mughannam and Reinke.^1^ The three cats in their case series had GT and calcaneal tendon avulsions with intact SDFT,^1^ consistent with Meutstege type IIc injuries.^6^ In the present study the only cat with persistent lameness at long-term evaluation had a Meutstege type I injury of the CCT. All other conservatively managed cats with available long-term outcome encountered type IIc injuries and were free of lameness at the time of long-term evaluation (Table 5).

A retrospective study in humans stated that surgical treatment of AT injuries was associated with a lower risk of re-rupture but a higher risk of complications such as infections, disturbed skin sensitivity and adhesions.^15^ However, owing to the low number of cats with available information on short- and long-term outcomes in the conservative treatment group, it was not possible to draw any conclusions in our study. A larger sample size of cats with conservatively treated CCT lesions compared with surgically treated lesions is necessary to evaluate if Meutstege type IIc lesions might be suitable for conservative treatment or surgical immobilisation of the tibiotarsal joint without primary tendon repair.

Only two cats showed re-rupture of the CCT. One of those cats had issues with the temporary transarticular immobilisation technique, which used a modified ESF type II frame. It is unknown when the ESF frame was removed by the local veterinarian, but the cat had an ESF PMMA frame breakage 14 days after initial surgery, followed by presentation with a re-rupture of the CCT 5 weeks later. There were a few different suture patterns and suture types used in the 55 surgically treated cats with AT injuries. Given that there were only two re-ruptures in the study population, it can be postulated that the suture pattern or suture type and strength are of secondary importance and that clinicians should focus on consequent postoperative transarticular immobilisation. In the three failed cases, the veterinarians employed combinations of different suture types. Unfortunately, the specific suture patterns were not recorded. The three-loop pulley is considered biomechanically superior to the locking loop.^17–19^ In our study, only 5.6% of cases were sutured with the three-loop pulley, and none of these cases encountered a tendon re-rupture.

Although many studies recommend postoperative transarticular immobilisation for approximately 6 weeks,^3,20,21^ clinicians should keep in mind that tendons need time to regain their normal breaking strength. After 6 weeks of immobilisation, 50% of normal tendon strength is restored and 79% of the normal breaking strain is achieved after 1 year.^22^ Strong immobilisation of the tendon can lead to delayed tendon healing because repetitive loading is required to stimulate collagen formation and fibre remodelling.^23,24^ Therefore, Schulz et al^25^ recommended another postoperative care regimen with a bi-valved full fibreglass cast from the toes to the proximal tibial region and dynamisation at 4–6 weeks by removing the cranial portion of the cast. At 6–8 weeks, they replaced the cast with a neoprene wrap and thermoplastic, and at 8–10 weeks they removed the whole external coaptation. Future studies should concentrate on long-term healing behaviour of the AT with different immobilisation techniques, which could be evaluated ultrasonographically.^26^ The selection of immobilisation technique depends on the patient, owner and environmental factors, as well as the surgeon’s preference. In a study with static loading of different immobilisation techniques, all provided similar immobilisation of the tibiotarsal joint. Transarticular ESF frames were not superior to calcaneotibial screws or cranial casts. However, evaluation of repetitive cyclic loading and forces that act on the tendon was not performed.^27^

In the short-term period, there was an overall complication rate of 41.3%, with 33.3% minor complications and 7.9% major complications. Cervi et al^3^ reported a total complication rate of 28.6%, with a relatively higher proportion of major complications (14.3%). They stated that all short-term complications were a result of the immobilisation technique. We confirm this observation because at least 75% of all minor complications and at least 40% of all major complications were attributable to the immobilisation technique. Complication rates of 39.5–46.5% have been reported in dogs after surgical treatment of AT injuries.^8,21^ The most common minor complications in our study were pin tract infections of ESF frames, or minor pin/screw loosening (ESF frame and calcaneotibial screw). There was a significant influence of external coaptation on the occurrence of minor complications. Of the 33.3% cats with minor complications, 42.9% had external coaptation, usually in addition to the primary type of transarticular immobilisation. Some of these cats (20%) developed skin sores due to bandage therapy (Table 4). This emphasises that there was a significant relationship between external coaptation and the occurrence of short-term complications, but the most commonly occurring minor complication was ESF-related pin tract infection (40%). As most minor complications were easy to manage, we cannot recommend one immobilisation technique over another, and decision-making should always depend on patient- and owner-specific circumstances and compliance. For example, some cats do not tolerate bandage therapy. In these cats a transarticular ESF frame might be the better option.Major complications could only be evaluated in a descriptive manner owing to the low occurrence rate. Except for one cat that suffered apophysiolysis of the tuber calcanei and another cat that experienced re-rupture of the CCT after implant removal, all other major complications could be linked to ESF frames or the calcaneotibial screw. The two re-ruptures of the tendon had preceding issues with the ESF frame and the other major complications were PMMA bar breakages with subsequent pin loosening or a calcaneal fracture after calcaneotibial screw placement. When considering complications, it is quite clear that most occur during the first 3 months after surgery: 94% of available cats had no further complications during the long-term evaluation period. Only three cats (6%) developed long-term complications, all of which were major. These cats had had complications during the short-term period. One cat even accounted for a total of four major complications during the short- and long-term periods.

Visual lameness analysis is very difficult in cats, and mild lameness can easily be overlooked during the orthopaedic examination.^28,29^ This problem might increase if the owners evaluate the lameness grades. Long-term evaluation was based on telephone calls with the referring veterinarians and an owner questionnaire. Therefore, the results might be biased by non-specialist judgements. The use of force plate analysis and measurement of ground reaction forces would have been more objective and could have detected mild residual hindlimb lameness in the study population.^28,29^ Evaluation with kinetic gait analysis was not possible. Hence, we decided to adopt the owner questionnaire used in a former study^3^ to make comparisons with the results of the present study easier.

The limitations to our study include the population size, which was too small for the statistical analysis of the influence of sex and type of treatment (surgical vs conservative) on complications and outcome. Further, the small number of major complications at the short- and long-term evaluations meant only descriptive statistical analysis was possible. The subjective evaluation of the long-term outcome through a questionnaire and the reliance on the owners may limit the results and conclusions of our study. Furthermore, owing to the retrospective nature of the study, not all cats were alive at the time of preparing the manuscript, and some information is based on memories of the owners. The questionnaire was translated into German and was not validated before use in our study. Generally, data evaluation was difficult owing to the use of different documentation software and different types of treatment of the participating clinics. All clinics were referral centres or teaching hospitals; therefore, short-term evaluation was usually available, but long-term evaluation and additional treatments were performed by the referring veterinarians, leading to a lack of information in the clinical records.

## Conclusions

The outcome after AT injuries in cats is generally good, with a very satisfying functional long-term outcome. The complication rates seem to be comparable to those in dogs, with a high proportion of minor complications due to the method of temporary immobilisation of the tarsal joint. ESF frames are more commonly involved in complications than other immobilisation options. Surgically treated cats had a slightly better long-term outcome than conservatively treated cats. However, proof of our hypothesis is not possible owing to the low number of conservatively treated cats with available long-term outcome information. Regarding the healing behaviour of tendons, an immobilisation method with staged dynamisation might be a promising approach for future studies.

## Supplemental Material

Table 1 Synopsis of patient data, injury specifications, therapy and outcome in 66 cats

Appendix Owner questionnaire
