# Supplementary material for: Retrospective multicentre evaluation of common calcaneal tendon injuries in 66 cats. Part 2: treatment, complications and outcomes
Source: J Feline Med Surg. 2023 Jan 27;25(1):1098612X221131224. doi: 10.1177/1098612X221131224 (PMC10812042; doi:10.1177/1098612X221131224)
Supplement: Table 1 [file sj-docx-1-jfm-10.1177_1098612X221131224.docx]

**Supplementary Material to:**

A retrospective multi-centre evaluation of treatment, complications, and outcome of common calcaneal tendon injuries in 66 cats

T. C. Häußler; M. Kornmayer; M. Scheich; A. Fischer; Christian J. Feichtenschlager; T. Rohwedder

Appendix Table 1: Synopsis of patient data, injury specifications, therapy, and outcome in 66 cats

| **Patient data** | | | | | **Injury specifications** | | | | **Therapy** | | | | **Outcome** | | | |
| --- | --- | --- | --- | --- | --- | --- | --- | --- | --- | --- | --- | --- | --- | --- | --- | --- |
| **Cat** | **Breed** | **Age**  **(years)** | **BW**  **(kg)** | **Gender** | **Type of injury** | **Type of trauma** | **Time to presentation (days)** | **Meutstege type** | **Type of therapy** | **Type of immobilisation** | **Duration of immobilisation (days)** | **Duration of additional immobilisation (days)** | **Short-term outcome** | **Long-term outcome** | **Questionnaire points**  **(out of 70)** | **Questionnaire percentage** |
| 1 | DSH | 10 | 6.6 | fs | 1 | 4 | 3 | IIc | 2 | 1 | 56 | 0 | 0 | 0 | 49 | 70.00 % |
| 2 | DSH | 9 | 5.3 | mc | 1 | 1 | 3 | IIc | 1 | 1 | 56 | 0 | 0 | 0 | 55 | 78.57 % |
| 3 | DSH | 9.8 | 4.2 | fs | 1 | 3 | 4 | IIc | 2 | 1 | 70 | 0 | 0 | 0 | 66 | 94.29 % |
| 4 | DSH | 0.8 | 2.7 | f | 2 | 2 | 0 | I | 2 | 1 | 42 | 14 | 1 | 0 | 64 | 91.43 % |
| 5 | DSH | 15 | 4.8 | mc | 1 | 1 | 1 | IIc | 2 | 1 | 48 | 0 | 0 | na | na | na |
| 6 | DSH | 1.3 | 3.2 | fs | 2 | 5 | 1 | IIc | 2 | 1 | 49 | 0 | 1 | 0 | 60 | 85.71 % |
| 7 | DSH | 11 | 5.5 | fs | 1 | 7 | 1 | IIc | 1 | 2 | 56 | 0 | 0 | na | na | na |
| 8 | DSH | 12 | 5.5 | fs | 1 | 7 | 0 | IIc | 2 | 1 | 49 | 0 | 0 | na | na | na |
| 9 | DSH | 9.2 | 6 | mc | 1 | 4 | 0 | IIa | 2 | 2 | 49 | 0 | 0 | 0 | 56 | 80.00 % |
| 10 | DSH | 13 | 4 | fs | 1 | 1 | 14 | IIc | 3 | 2 | 42 | 0 | 0 | 0 | 61 | 87.14 % |
| 11 | DSH | 6 | 5 | mc | 1 | 1 | 10 | I | 2 | 2 | 56 | 0 | 0 | 0 | 66 | 94.29 % |
| 12 | DSH | 16 | 4.4 | fs | 1 | 1 | 1 | IIc | 1 | 2 | 56 | 0 | 0 | 0 | 62 | 88.57 % |
| 13 | DSH | 2.3 | 5 | mc | 2 | 2 | 10 | I | 2 | 1 | 46 | 0 | 1 | na | na | na |
| 14 | DSH | 6.9 | 4.6 | mc | 1 | 3 | 0 | I | 2 | 1 | 37 | 0 | 1 | 0 | 66 | 94.29 % |
| 15 | DSH | 4.7 | 3.9 | mc | 1 | 1 | 21 | IIa | 2 | 1 | 36 | 0 | 0 | 0 | 66 | 94.29 % |
| 16 | DSH | 0.7 | 3.7 | mc | 1 | 1 | 14 | I | 2 | 1 | 56 | 0 | 1 | na | na | na |
| 17 | DSH | 3.5 | 6.2 | mc | 2 | 2 | 1 | I | 2 | 1 | 42 | 0 | 2 | na | na | na |
| 18 | S | 1.8 | 3.7 | fs | 1 | 6 | 0 | I | 2 | 1 | 43 | 0 | 2 | 0 | 58 | 82.86 % |
| 19 | TA | 14 | 3.5 | fs | 1 | 7 | 35 | IIc | 2 | 3 | 36 | 0 | 2 | 2 | 30 | 42.86 % |
| 20 | DSH | 7.6 | 4.3 | mc | 1 | 1 | 8 | IIc | 2 | 1 | 40 | 0 | 0 | 0 | 64 | 91.43 % |
| 21 | DSH | 0.7 | 3.5 | mc | 2 | 3 | 2 | IIc | 2 | 1 | 28 | 0 | 0 | na | na | na |
| 22 | DSH | 9.8 | 4.6 | f | 1 | 1 | 1 | I | 1 | 1 | 38 | 0 | 2 | 2 | 52 | 74.29 % |
| 23 | DSH | 0.5 | 3 | mc | 1 | 1 | 1 | IIc | 2 | 1 | na | na | na | na | na | na |
| 24 | DSH | 16 | 4 | fs | 2 | 8 | 4 | IIc | 2 | 1 | 42 | 28 | 0 | 0 | 63 | 90.00 % |
| 25 | DSH | 2.8 | 4.5 | fs | 1 | 1 | 75 | IIa | 2 | 1 | 42 | 14 | 0 | 2 | 46 | 65.71 % |
| 26 | A | 10 | 4.9 | fs | 2 | 3 | 1 | I | 2 | 1 | 42 | 35 | 0 | na | na | na |
| 27 | BSH | 13 | 3.5 | fs | 1 | 1 | 10 | III | 1 | 3 | 42 | 0 | 0 | na | na | na |
| 28 | DSH | 7.2 | 4 | mc | 2 | 2 | 1 | I | 2 | 1 | 42 | 14 | 0 | 0 | 70 | 100.00 % |
| 29 | DSH | 13 | 4.5 | mc | 1 | 6 | 6 | IIc | 1 | 3 | 28 | 0 | 0 | na | na | na |
| 30 | DSH | 11 | 9 | mc | 1 | 7 | 28 | IIb | 2 | 1 | 84 | 14 | 1 | 1 | 46 | 65.71 % |
| 31 | DSH | 10 | 5.8 | mc | 1 | 7 | 10 | IIc | 1 | 3 | 42 | 0 | 0 | 0 | 57 | 81.43 % |
| 32 | DSH | 12 | 4.7 | fs | 1 | 7 | 1 | IIc | 2 | 1 | 42 | 14 | 0 | 0 | 60 | 85.71 % |
| 33 | DSH | 14 | 4.7 | f | 1 | 1 | 1 | IIc | 2 | 1 | 56 | 2 | 1 | 0 | 50 | 71.43 % |
| 34 | DSH | 3 | 4.5 | f | 1 | 1 | 7 | I | 2 | 4 | 42 | 0 | 1 | 0 | 59 | 84.29 % |
| 35 | DSH | 14 | 6 | mc | 1 | 7 | 2 | na | 1 | na | na | 0 | na | na | na | na |
| 36 | DSH | 0.7 | 1.5 | f | 1 | 6 | 7 | I | 2 | 4 | 42 | 0 | 2 | 0 | 64 | 91.43 % |
| 37 | DSH | 11 | 4.8 | fs | 1 | 1 | 5 | I | 2 | 4 | 28 | 10 | 2 | 0 | 61 | 87.14 % |
| 38 | DSH | 0.5 | 3 | mc | 1 | 7 | 4 | I | 2 | 5 | 2 | 2 | 0 | 0 | 60 | 85.71 % |
| 39 | DSH | 4 | 4.5 | fs | 2 | 2 | 7 | I | 2 | 4 | 42 | 0 | na | na | na | na |
| 40 | DSH | 12 | 5.1 | fs | 1 | 7 | 9 | IIc | 2 | 4 | 42 | 0 | 4 | na | na | na |
| 41 | DSH | 4 | 3 | fs | 1 | 1 | 3 | IIc | 2 | 4 | 49 | 0 | 2 | na | na | na |
| 42 | DSH | 1.5 | 5.2 | fs | 2 | 2 | 1 | I | 2 | 4 | 63 | 0 | 0 | 0 | 58 | 82.86 % |
| 43 | DSH | 10 | 4.2 | fs | 1 | 1 | 0 | IIc | 2 | 1 | na | 0 | na | na | na | na |
| 44 | DSH | 11 | 4.5 | fs | 1 | 7 | 20 | IIc | 2 | 4 | 56 | 0 | 0 | 0 | 63 | 90.00 % |
| 45 | DSH | 12 | 4.5 | fs | 1 | 7 | 5 | IIc | 2 | 4 | 42 | 0 | 0 | 0 | 63 | 90.00 % |
| 46 | DSH | 3 | 4.9 | fs | 2 | 2 | 1 | I | 2 | 4 | 42 | 0 | 0 | 0 | 64 | 91.43 % |
| 47 | DSH | 6 | 3.8 | fs | 2 | 1 | 1 | I | 2 | 4 | 42 | 0 | na | 0 | 58 | 82.86 % |
| 48 | DSH | 5 | 5 | fs | 1 | 1 | 3 | IIc | 2 | 4 | 42 | 0 | 0 | 0 | 61 | 87.14 % |
| 49 | DSH | 5 | 3.8 | mc | 1 | 1 | 5 | IIc | 2 | 4 | 56 | 0 | 0 | 0 | 56 | 80.00 % |
| 50 | DSH | 10 | 3.6 | fs | 2 | 2 | 0 | I | 2 | 4 | 42 | 0 | na | 0 | 63 | 90.00 % |
| 51 | DSH | 12 | 5.3 | mc | 1 | 1 | 2 | IIc | 2 | 4 | 77 | 0 | 0 | 0 | 64 | 91.43 % |
| 52 | BSH | 8 | 8.5 | mc | 1 | 7 | 23 | IIc | 1 | na | 70 | 0 | 2 | 0 | 63 | 90.00 % |
| 53 | DSH | 0.6 | 3.9 | mc | 2 | 2 | 0 | I | 2 | 4 | 56 | 0 | 0 | 0 | 59 | 84.29 % |
| 54 | BSH | 2 | 4.8 | mc | 2 | 2 | 1 | I | 2 | 4 | 98 | 0 | 2 | 2 | 48 | 68.57 % |
| 55 | DSH | 4 | 4.5 | mc | 1 | 7 | 2 | IIc | 1 | na | na | na | na | 0 | 51 | 72.86 % |
| 56 | DSH | 2 | 3 | fs | 2 | 2 | 0 | I | 2 | 4 | 84 | 0 | 0 | 2 | 45 | 64.29 % |
| 57 | DSH | 5 | 5.2 | mc | 2 | 2 | 0 | I | 2 | 4 | 56 | 0 | 0 | 0 | 67 | 95.71 % |
| 58 | DSH | 12 | 6.4 | mc | 2 | 1 | 5 | IIc | 2 | 4 | 49 | 0 | 0 | 0 | 63 | 90.00 % |
| 59 | DSH | 10 | 4.2 | fs | 1 | 1 | 18 | I | 2 | 4 | 63 | 0 | 0 | 0 | 63 | 90.00 % |
| 60 | DSH | 8 | 6 | fs | 2 | 2 | 16 | I | 2 | 2 | 84 | 0 | 0 | 0 | 65 | 92.86 % |
| 61 | DSH | 13 | 5.3 | fs | 1 | 1 | 1 | IIc | 2 | 2 | 28 | 0 | 0 | 0 | 68 | 97.14 % |
| 62 | DSH | 15 | 6.2 | mc | 1 | 7 | 2 | IIc | 2 | 2 | 42 | 0 | 0 | 0 | 66 | 94.29 % |
| 63 | DSH | 6.3 | 4.5 | fs | 1 | 1 | 14 | I | 2 | 2 | 35 | 0 | 0 | 0 | 63 | 90.00 % |
| 64 | DSH | 10 | 4.2 | fs | 1 | 1 | 1 | IIc | 2 | 3 | 42 | 0 | 0 | 0 | 63 | 90.00 % |
| 65 | DSH | 1.4 | 3.6 | fs | 1 | 1 | 14 | IIc | 2 | 2 | 42 | 0 | 0 | 0 | 66 | 94.29 % |
| 66 | B | 1.8 | 3.4 | fs | 2 | 1 | 185 | IIc | 2 | 2 | 42 | 0 | 0 | 0 | 60 | 85.71 % |

**Abbreviations**

- na = not available
- Breed: DSH = Domestic Shorthair, BSH = British Shorthair, TA = Turkish Angora, S = Siamese, A = Abyssinian, B = Bengal
- Sex: fs = female spayed, f = female intact, mc = male castrated
- Type of injury: 1 = closed injury, 2 = open injury
- Type of trauma: 1 = unknown, 2 = laceration injury, 3 = high rise trauma, 4 = during running/chasing, 5 = car accident, 6 = blunt trauma (catching/pinching injury), 7 = minor trauma (jumping, playing), 8 = biting injury
- Meutstege type: 1 = type I, 2 = type IIa, 3 = type IIb, 4 = type IIc, 5 = type III, na = not available
- Type of therapy: 1 = surgically, 2 = conservatively (only immobilisation, no primary surgical tendon repair)
- Type of immobilisation: 1 = transarticular external skeletal fixator (ESF) type II, 2 = transarticular ESF type II modified, 3 = cast and external coaptation, 4 = calcaneotibial screw, 5 = tendon plating with external coaptation
- Short-term outcome / Long-term outcome: 0 = no lameness, 1 = grade 1/4 lameness, 2 = grade 2/4 lameness, 3 = grade 3/4 lameness, 4 = grade 4/4 lameness
