# Supplementary material for: Retrospective multicentre evaluation of common calcaneal tendon injuries in 66 cats. Part 2: treatment, complications and outcomes
Source: J Feline Med Surg. 2023 Jan 27;25(1):1098612X221131224. doi: 10.1177/1098612X221131224 (PMC10812042; doi:10.1177/1098612X221131224)
Supplement: Appendix [file sj-docx-2-jfm-10.1177_1098612X221131224.docx]

**Questionnaire – Achilles tendon injuries in cats**

by Cervi et al. 2010 (VCOT), slightly modified

Owner: _______________________ Cat’s name: _________________

Thank you very much in advance for the completion of the questionnaire.

Please read the instructions first

**Instructions**

- Please answer to the questions by encircling the most accurate statement.
- Multiple circles are not allowed.

1

| How would you describe your overall assessment of your cat’s recovery? | | | | |
| --- | --- | --- | --- | --- |
| 1  Poor | 2  Below average | 3  Average | 4  Good | 5  Excellent |

2

| How has your cat’s attitude been since surgery? | | | | |
| --- | --- | --- | --- | --- |
| 1  Negative | 2  Mildly negative | 3  No change | 4  Mildly positive | 5  Positive |

3

| Has your cat changes the amount of activities since surgery? | | | | |
| --- | --- | --- | --- | --- |
| 1  Much less | 2  Slightly less | 3  No change | 4  Slightly more | 5  Much more |

4

| How often did your cat play before the injury? | | | | |
| --- | --- | --- | --- | --- |
| 1  Never | 2  Rarely | 3  Sometimes | 4  Often | 5  Very often |

| How often does your cat play now after surgery? | | | | |
| --- | --- | --- | --- | --- |
| 1  Never | 2  Rarely | 3  Sometimes | 4  Often | 5  Very often |

5

6

| Does your cat show joint stiffness when rising for the day or after a long rest? | | | | |
| --- | --- | --- | --- | --- |
| 1  Always | 2  Often | 3  Sometimes | 4  Rarely | 5  Never |

7

| Does your cat show any joint stiffness at the end of the day and post-activities? | | | | |
| --- | --- | --- | --- | --- |
| 1  Always | 2  Often | 3  Sometimes | 4  Rarely | 5  Never |

8

| Does your cat show any lameness of the operated limb? | | | | |
| --- | --- | --- | --- | --- |
| 1  Always | 2  Often | 3  Sometimes | 4  Rarely | 5  Never |

9

| Does your cat indicate pain when turning suddenly at walk? | | | | |
| --- | --- | --- | --- | --- |
| 1  Always | 2  Often | 3  Sometimes | 4  Rarely | 5  Never |

10

| Does your cat walk normally? | | | | |
| --- | --- | --- | --- | --- |
| 1  Always | 2  Often | 3  Sometimes | 4  Rarely | 5  Never |

11

| Is your cat able to jump to similar heights as before surgery? | | | | |
| --- | --- | --- | --- | --- |
| 1  Same | 2  Little less | 3  Less | 4  Much less | 5  Not at all |

12

| Is your cat reluctant to play for as long as usual? | | | | |
| --- | --- | --- | --- | --- |
| 1  Same | 2  Little less | 3  Less | 4  Much less | 5  Won’t play at all |

13

| Does your cat have trouble rising from a resting position? | | | | |
| --- | --- | --- | --- | --- |
| 1  Always | 2  Often | 3  Sometimes | 4  Rarely | 5  Never |

14

| Does your cat have difficulties climbing or descending stairs? | | | | |
| --- | --- | --- | --- | --- |
| 1  Always | 2  Often | 3  Sometimes | 4  Rarely | 5  Never |

15

| Does your cat have difficulties running, walking, jumping? | | | | |
| --- | --- | --- | --- | --- |
| 1  Always | 2  Often | 3  Sometimes | 4  Rarely | 5  Never |
